# Assessment of night eating syndrome and circadian-related characteristics among university students of Rawalpindi and Islamabad

**DOI:** 10.3389/fpubh.2026.1932196

**Published:** 2026-10-12

**Authors:** Muqadsa Arshad, Humaira Mahmood, Abdul Momin Rizwan Ahmad, Abdullah Ijaz, Tehreem Zainab, Yasir Waheed, Saleh A. Alsanie

**Affiliations:** 1 Department of Public Health, Armed Forces Postgraduate Medical Institute (AFPGMI), National University of Medical Sciences (NUMS), Rawalpindi, Pakistan; 2 School of Public Health, Health Services Academy, Islamabad, Pakistan; 3 Department of Human Nutrition and Dietetics, NUST School of Health Sciences, National University of Sciences and Technology (NUST), Islamabad, Pakistan; 4 Department of Health Sciences, University of York, York, United Kingdom; 5 NUST School of Health Sciences, National University of Sciences and Technology (NUST), Islamabad, Pakistan; 6 Irfan Suat Gunsel Operational Research Institute, Near East University, Nicosia, Mersin, Türkiye; 7 Faculty of Health Sciences, Széchenyi István University, Győr, Hungary; 8 Department of Health Sciences, VIZJA University, Warsaw, Poland; 9 Department of Basic Health Sciences, College of Applied Medical Sciences, Qassim University, Buraydah, Saudi Arabia; 10 Department of Clinical Nutrition, Medical City, Qassim University, Buraydah, Saudi Arabia

**Keywords:** circadian-related characteristics, night eating syndrome, non-communicable diseases, university students, young adults

## Abstract

**Introduction:**

Non-communicable diseases are associated with lifestyle behaviors such as diet, meal timing, levels of stress, quality of sleep, and disruption of circadian rhythms. Night Eating Syndrome (NES) is an abnormal eating behavior characterized by eating at night and/or eating after the last meal of the day. NES may be associated with sleep disturbances and/or circadian-related characteristics. University students are particularly susceptible to the development of NES due to academic stress, irregular schedules, increased exposure to screens, and late nights.

**Objective:**

This study aimed to determine the prevalence and severity of Night Eating Syndrome and to examine the relationships between Night Eating Syndrome and sleep-related characteristics, circadian-related characteristics (diurnal preference), and mood among university students in Rawalpindi and Islamabad, Pakistan.

**Methods:**

A cross-sectional analytical study was conducted on 335 undergraduate university students at four universities in Rawalpindi and Islamabad using a multistage sampling method. Data were collected using structured questionnaires including sociodemographic questions, the Night Eating Diagnostic Questionnaire, and the Sleep, Circadian Rhythm, and Mood questionnaire. Data analysis was performed using SPSS Version 27. Descriptive statistics, one-way ANOVA, Welch’s ANOVA with Games–Howell post-hoc comparisons, multiple linear regression, Fisher–Freeman–Halton exact test, and Pearson chi-square test were used, with significance set at *p* < 0.05.

**Results:**

The Prevalence of Night Eating Syndrome was 6% (*n* = 18; 95% CI: 3.2–8.4%). Severity-based classification showed that 51.0% had normal scores, 26.3% had mild symptoms, 17.3% had moderate symptoms, and 6% met the NEDQ criteria for NES. There were significant differences between NES severity groups in Morningness and Good Sleep scores (*p* < 0.001), while there were no significant differences in Depressed Mood scores (*p* = 0.159). In adjusted regression models, NES status was significantly associated with Good Sleep scores, while sex was associated with Morningness and BMI was associated with Depressed Mood. BMI category was significantly associated with NES status (Fisher–Freeman–Halton exact test, *p* = 0.002) and NES severity (*p* < 0.001).

**Conclusion:**

Night Eating Syndrome and related symptoms were present among university students, with increasing symptom severity associated with differences in Morningness and Good Sleep scores, while Depressed Mood did not differ significantly across severity categories. These findings suggest the possible significance of health promotion initiatives conducted through universities emphasizing good sleep hygiene, regular meal time and early identification of night eating symptoms.

## 1 Introduction

Non-communicable diseases (NCDs) continue to be a major public health burden and one of the most common causes of morbidity and death globally. NCDs led to over 41 million deaths worldwide, or approximately three-quarters of all fatalities, according to the Global Burden of Disease Study 2019 (1). NCDs have a major effect on disability, healthcare costs, reduced efficiency, and a lower quality of life in addition to mortality. Eating disorders have become increasingly recognized as major but comparatively unaddressed public health issues within this larger burden. According to recent estimates, eating disorders contributed to the population-level illness burden by accounting for almost 6.6 million disability-adjusted life years worldwide (2). Rising trends in NCDs were attributed mainly to lifestyle-related risk factors including unhealthy diet, physical inactivity, psychological stress, altered sleep patterns and dysregulated biological clocks (3, 4).

Rapid urbanization, lifestyle modification, and technology have influenced dietary and daily activity patterns. The nutrition transition has been associated with increased consumption of energy-dense foods, sedentary lifestyle, inappropriate meal times, and lack of sleep (5). These lifestyle changes may be associated with differences in sleep schedules, screen exposure, and eating patterns, which may in turn relate to circadian-related behavioral characteristics. The understanding of the relationship between meal timing, sleep, and circadian-related characteristics is therefore increasingly important in the context of healthy eating behaviors.

The circadian rhythm is an endogenously generated biological process that controls physiological and behavioral activities in an approximate 24-h cycle. Circadian rhythms are controlled by the suprachiasmatic nucleus, which acts as the master biological clock coordinating all peripheral clocks in the metabolically active organs like the liver, pancreas, GI tract, fat tissues, and muscles (6, 7) The role of circadian rhythms is to coordinate sleep–wake cycles, hormonal release, feeding behavior, glucose metabolism, and energy homeostasis. Zeitgebers in the form of light exposure and meal timing provide environmental information for the synchronization of biological clocks with the environment (8).

Circadian rhythm disruption has been associated with adverse metabolic health outcomes and an increased risk of several chronic illnesses. Circadian misalignment resulting from delayed sleep schedules, irregular sleep patterns, social jetlag, and variable meal time has been associated with obesity, metabolic syndrome, insulin resistance, cardiovascular disease, and type 2 diabetes mellitus (T2DM) (9, 10). Furthermore, appetite hormones including ghrelin, leptin, insulin, and cortisol, which are known to influence hunger, satiety, and food intake, may be regulated downstream by circadian disturbances (11). Therefore, the relationship between lifestyle patterns, metabolic and nutritional health, and circadian-related behavioral characteristics has received increasing attention in health research.

The concept of chrononutrition emphasizes the relevance of meal timing in metabolic control. Unlike traditional nutritional methods, which are primarily concerned with dietary quantity and quality, chrononutrition focuses on food intake time and its connection with the body’s circadian clock (12). Food consumption during physiologically unsuitable times appears to affect metabolic control, increasing the risk of obesity and other metabolic abnormalities (13, 14). These findings have increased scientific interest in eating behaviors characterized by delayed food intake, particularly Night Eating Syndrome (NES).

Night Eating Syndrome was first described by Stunkard in 1955 and is characterized by a delayed pattern of food consumption involving increased intake during the evening or nighttime hours (15). Evening hyperphagia, nocturnal eating episodes, morning anorexia, weariness, and negative mood in the evening are commonly associated with the syndrome (16). NES is distinguished from binge eating disorder, which is characterized by excessive food consumption and a lack of control, by the time of food intake and its connection to sleep and circadian rhythm abnormalities (17). NES is presently categorized under Other Specified Feeding and Eating Disorders in the DSM-V (Diagnostic and Statistical Manual of Mental Disorders), Fifth Edition, and is also known to be linked with obesity, poor sleep, psychological distress, and reduced quality of life (18, 19).

There is growing evidence that the association between NES and sleep regulation and circadian rhythms is well-founded based on earlier research. Delayed eating and changes in the timing of energy, carbohydrates, and fat intake have been reported in cases of NES (20). Earlier research has also reported differences in hormone production, appetite regulation, and sleep rhythms among individuals with NES, supporting an association between NES behavior, sleep, and circadian-related processes (21, 22). This evidence underscores the importance of examining sleep and circadian-related behavioral characteristics when studying NES.

University students may be more prone to disturbed patterns of eating and sleeping habits due to academic pressures, varying schedules, social events, and heavy dependence on technology (23, 24). University students suffer from social jet lag and poor sleep, and both these factors are known to lead to negative effects on their health (25, 26). Such factors may be associated with differences in sleep–wake patterns and eating behaviors among young people (44).

Several studies conducted among university students have highlighted the existence of Night Eating Syndrome, as well as its link to sleep problems, psychological problems, and poor eating habits. Students with irregular sleep patterns and academic stress are more likely to participate in night eating practices and have poorer mental health outcomes, according to data from China, Palestine, Bangladesh, Malaysia, and Turkey (27–31). Similarly, systematic evidence suggests that university students may experience a higher prevalence of NES compared with the general population, highlighting the vulnerability of this age group to irregular eating patterns, sleep disturbances, and circadian-related behavioral characteristics (32, 33). Recent study has linked NES among college students to depression, anxiety, poor sleep, food addiction, and poor academic performance (34–36).

Although there is not enough evidence available about South Asian countries despite the global research focus on Night Eating Syndrome and circadian rhythm, most of the literature found about the topic is in relation to Western people, adolescents, or clinical settings (17). Several factors may influence eating and sleep-related patterns among university students in Pakistan, including late-night eating, inconsistent class schedules, hostel residence, and increased exposure to technology. Even though there have been some studies related to eating disorders of Pakistani university students, there is no information about the prevalence of Night Eating Syndrome and association with sleep or circadian rhythms including diurnal preference among them (37, 38). This gap in locally relevant evidence highlights the need for further research among university students in Pakistan.

Thus, the objective of the present study was to determine the prevalence and severity of Night Eating Syndrome and to examine the relationships between Night Eating Syndrome and sleep-related characteristics, circadian-related characteristics (diurnal preference), and mood among university students in Rawalpindi and Islamabad, Pakistan.

## 2 Materials and methods

### 2.1 Study design, setting, and participants

The study was a cross-sectional analytical study conducted among undergraduate students of Allied Health Sciences enrolled in selected universities of Rawalpindi and Islamabad, Pakistan. The study was carried out for a period of 8 months, from November 2025 to June 2026, after approval of the study protocol by the Institutional Review Board of Armed Forces Postgraduate Medical Institute and administrative permission from participating universities. The participating institutions were Pir Mehr Ali Shah Arid Agriculture University Rawalpindi, Health Services Academy Islamabad, International Islamic University Islamabad, and Shifa Tameer-e-Millat University.

The study population included undergraduate students aged between 18 and 24 years who were enrolled in Allied Health Sciences programs and in the fourth to eighth semesters. Students from the fourth to eighth semesters were included because these were the semesters available for data collection at the participating institutions during the study period. To be included, students had to be present at the time of data collection and give written informed consent. Individuals with a self-reported or clinical diagnosis of a psychiatric disorder, eating disorder, or sleep disorder were excluded because of the possibility that these disorders could influence night eating behavior and circadian rhythm-related characteristics. Students on medications or therapeutic diets that could have a significant impact on food intake or sleep were also excluded. Students who did not give informed consent and those who were absent during data collection were excluded.

### 2.2 Sample size and sampling procedure

The required sample size was calculated using the standard formula for estimating a single population proportion:

$$\frac{n_{0}=Z^{2}P(1-P)}{d^{2}}$$

where *Z* = 1.96 for a 95% confidence level, *p* = 0.178, representing the prevalence estimate reported among university students in Bangladesh (39), and d = 0.05, representing 5% absolute precision.

Substituting the values into the formula:

$$\frac{n_{0}={\left(1.96\right)}^{2}\times 0.178\times (1-0.178)}{{\left(0.05\right)}^{2}}$$

$$n_{0}=224.84\approx 225$$

To account for potential non-response and incomplete or invalid responses, a 25% inflation was applied to the initial sample size. The adjusted sample size was calculated using:

$$\frac{n=n_{0}}{\left(1-r\right)}$$

where *r* = 0.25 represents the anticipated non-response rate.

$$\frac{n=225}{\left(1-0.25\right)}$$

n=300

Therefore, the minimum required sample size was 300 participants. To ensure adequate data for analysis, a total of 335 undergraduate students were included in the study.

A multi-stage sampling technique was used. In the first stage, universities with Allied Health Sciences programs in Rawalpindi and Islamabad were identified from the website of the Higher Education Commission of Pakistan (HEC), since the population targeted consisted of undergraduate students pursuing studies in Allied Health Sciences programs. Relevant Allied Health Sciences departments of these universities were further identified. Permission was taken from the eligible universities, out of which four universities who granted administrative permission were selected to take part in this study. In the second stage, lists of eligible students enrolled in semesters four to eight were obtained from the respective Allied Health Sciences departments of the four participating universities. A target of 84 participants was set for each university, resulting in an approximately equal allocation across the four institutions. Within each sampling frame, systematic random sampling was used. The sampling interval (k) was calculated using the formula k = N/n, where N represented the total number of eligible students in the sampling frame and n represented the required number of participants from that sampling frame. The first participant was selected using a lottery method, and subsequent participants were selected at every kth interval until the required sample was reached. The selected semesters represented the semesters available during the data collection period.

### 2.3 Data collection

Data were collected using structured, self-administered questionnaires, which was approved by the ethics committee and the institution. First, prior to conducting the data collection, all the participants were informed about the study, its voluntariness, confidentiality of information and the possibility to withdraw from the study at any point. All participants provided their written consent before participation.

The sociodemographic variables were age, sex, type of program, academic year, marital status, living arrangement, and occupation status. The participants were requested to report their height and body weight. They were encouraged to report current, accurate measurements, preferably without footwear and wearing light clothes. Body Mass Index was calculated as the ratio of weight in kilograms and height in meters squared. BMI categories were classified using Asian-specific cut-off points as follows: underweight (<18.5 kg/m^2^), normal weight (18.5–22.9 kg/m^2^), overweight (23–24.9 kg/m^2^), obese class I (25–29.9 kg/m^2^), and obese class II (≥30 kg/m^2^).

### 2.4 Assessment of night eating syndrome

Night Eating Syndrome was assessed using the Night Eating Diagnostic Questionnaire (NEDQ), a 21-item instrument validated by Nolan and Geliebter (39). The questionnaire assesses features of night eating, including evening hyperphagia, nocturnal eating, morning anorexia, insomnia, awareness and recall of night eating, evening mood, and distress or impairment related to night eating behaviors. According to the NEDQ scoring criteria, full NES requires increased evening or nocturnal food intake, awareness and recall of evening or nocturnal eating episodes, at least three of five associated features, significant distress or impairment, and maintenance of the disordered eating pattern for at least 3 months. Participants were classified as either meeting or not meeting the NEDQ criteria for full NES based on these criteria. In addition, the hierarchical scoring system described in the NEDQ was used to classify participants into normal, mild, moderate, and full NES categories. Participants who did not meet any of the hierarchical criteria were classified as normal. Participants meeting one criterion from Criterion I, but not the criteria for moderate or full NES, were classified as mild. Participants meeting one criterion from Criterion I and at least three of the five features under Criterion III, but not the full NES criteria, were classified as moderate. Participants meeting the full NEDQ criteria, including Criterion I, at least three of the five features under Criterion III, and the required distress/impairment and duration criteria, were classified as full NES. The mild and moderate categories represented increasing levels of night-eating symptoms and did not indicate that participants met the full NEDQ criteria for NES.

### 2.5 Assessment of circadian rhythm-related characteristics

In the present study, circadian rhythm-related characteristics were operationalized primarily through the Morningness subscale of the Sleep, Circadian Rhythms and Mood (SCRAM) questionnaire, which reflects individual differences in diurnal preference. This represents a behavioral circadian-related characteristic and does not constitute a clinical diagnosis of a circadian rhythm disorder or a direct physiological measurement of circadian function. The SCRAM questionnaire was used to assess circadian-related, sleep-related, and mood-related characteristics (40). It consists of three subscales: Morningness, Good Sleep, and Depressed Mood, with five items in each subscale. Each item was scored on a six-point Likert scale ranging from 1 (strongly disagree) to 6 (strongly agree). One item from each subscale was reverse-scored according to the original SCRAM scoring method. Scores were obtained by summing the five items within each subscale after reverse scoring, giving a possible score range of 5–30 for each subscale, with higher scores indicating greater strength of the respective construct.

### 2.6 Statistical analysis

Data analysis was done through entering, cleaning, and analysis of data using SPSS 27.0 software. Continuous variables such as age, BMI, and SCRAM subscales were reported in mean ± standard deviation. Frequency and percentage were used for reporting categorical variables. The normality distribution of continuous variables was checked through measures of skewness and kurtosis. Since the variables of interest were normally distributed, parametric statistical methods were used. For comparison of SCRAM subscale scores across NES severity categories, one-way analysis of variance (ANOVA) was conducted. Homogeneity of variance was assessed, and Welch’s ANOVA was used when this assumption was violated, whereas conventional one-way ANOVA was used when the relevant assumptions were met. Games–Howell post-hoc tests were performed for pairwise comparisons following significant overall group differences. Mean differences, 95% confidence intervals, and adjusted *p*-values were reported. Effect sizes were reported using eta-squared (η^2^) with 95% confidence intervals. Multiple linear regression analyses were performed for Good Sleep, Morningness, and Depressed Mood scores, with NES status, sex, and BMI included as predictors. NES status, sex, and BMI were selected because they were the main variables of interest in the study and were available for inclusion in the adjusted models. Regression coefficients (*B*), standardized beta coefficients (*β*), *p*-values, *R*^2^, adjusted *R*^2^, F-statistics, and overall model *p*-values were reported. NES status was coded as 0 = No NES and 1 = NES, with No NES as the reference category. Sex was coded as 0 = Male and 1 = Female, with Male as the reference category. Other potentially relevant confounding variables were not included in the regression models because they were not available for the adjusted analysis. The Fisher–Freeman–Halton exact test was used to assess the association between BMI categories and NES status because of sparse expected cell counts. Pearson’s chi-square test was used to assess the association between BMI categories and NES severity, and the likelihood-ratio chi-square statistic was also reported. The likelihood-ratio chi-square statistic was also reported for the association between BMI category and NES severity.

## 3 Results

### 3.1 Sociodemographic characteristics of participants

A total of 335 undergraduate students were considered in the final analysis. The mean age of the subjects was 21.97 ± 1.15 years. The mean height was 62.77 ± 3.33 inches, the mean weight was 49.45 ± 8.41 kg, and the mean Body Mass Index (BMI) was 19.43 ± 3.00 kg/m^2^.

Table 1 provides information on the sociodemographic profile of the participants. Of these, most were females (*n* = 270, 80.6%), and 65 (19.4%) were males. As far as the study program goes, the majority of the participants belonged to BS Public Health (*n* = 97, 29.0%), BS Psychology (*n* = 78, 23.3%), BS Human Nutrition and Dietetics (*n* = 58, 17.3%), BS Medical Laboratory Technology (*n* = 58, 17.3%), and BS Doctor of Physical Therapy (*n* = 44, 13.1%). Almost 50% of the participants were from the third-year level (*n* = 176, 52.5%). Majority of the respondents were singles (*n* = 319, 95.2%), lived with family members (*n* = 184, 54.9%), and were unemployed (*n* = 304, 90.7%). Regarding BMI, 192 (57.3%) participants were underweight, 110 (32.8%) had normal weight, 16 (4.8%) were overweight, 13 (3.9%) had obesity class I, and 4 (1.2%) had obesity class II.

**Table 1:** Sociodemographic characteristics of participants (*n* = 335).

Variable		Frequency (N)	Percentage (%)
Sex	Male	65	19.4
	Female	270	80.6
	Total	335	100%
Study program	BS Public Health	97	29.0
	BS HND	58	17.3
	BS MLT	58	17.3
	BS DPT	44	13.1
	BS Psychology	78	23.3
	Total	335	100%
Study year	2nd Year	104	31.0
	3rd year	176	52.5
	4th year	55	16.4
	Total	335	100%
Marital status	Single	319	95.2
	Married	16	4.8
	Total	335	100%
Living status	Hostel	151	45.1
	With family	184	54.9
	Total	335	100%
Employment status	No	304	90.7
	Part time	21	6.3
	Full time	10	3.0
BMI category	Underweight (<18.5 kg/m^2^)	192	57.3
	Normal weight (18.5–22.9 kg/m^2^)	110	32.8
	Overweight (23–24.9 kg/m^2^)	16	4.8
	Obese class I (25–29.9 kg/m^2^)	13	3.9
	Obese class II (≥30 kg/m^2^)	4	1.2
	Total	335	100%

### 3.2 Prevalence and severity of night eating syndrome

The severity categories of Night Eating Syndrome (NES) among the participants are shown in Figure 1. Using the Night Eating Diagnostic Questionnaire (NEDQ), 18 participants met the NEDQ criteria for NES, which is equivalent to 6% of the sample (95% confidence interval: 3.4–8.3%). The other 317 subjects (94%) did not meet the NEDQ criteria for NES. Among them, 171 participants (51.0%) were categorized as normal, 88 (26.3%) had mild symptoms, 58 (17.3%) had moderate symptoms, and 18 (6.0%) met the criteria for full NES (see Table 2).

**Figure 1:**
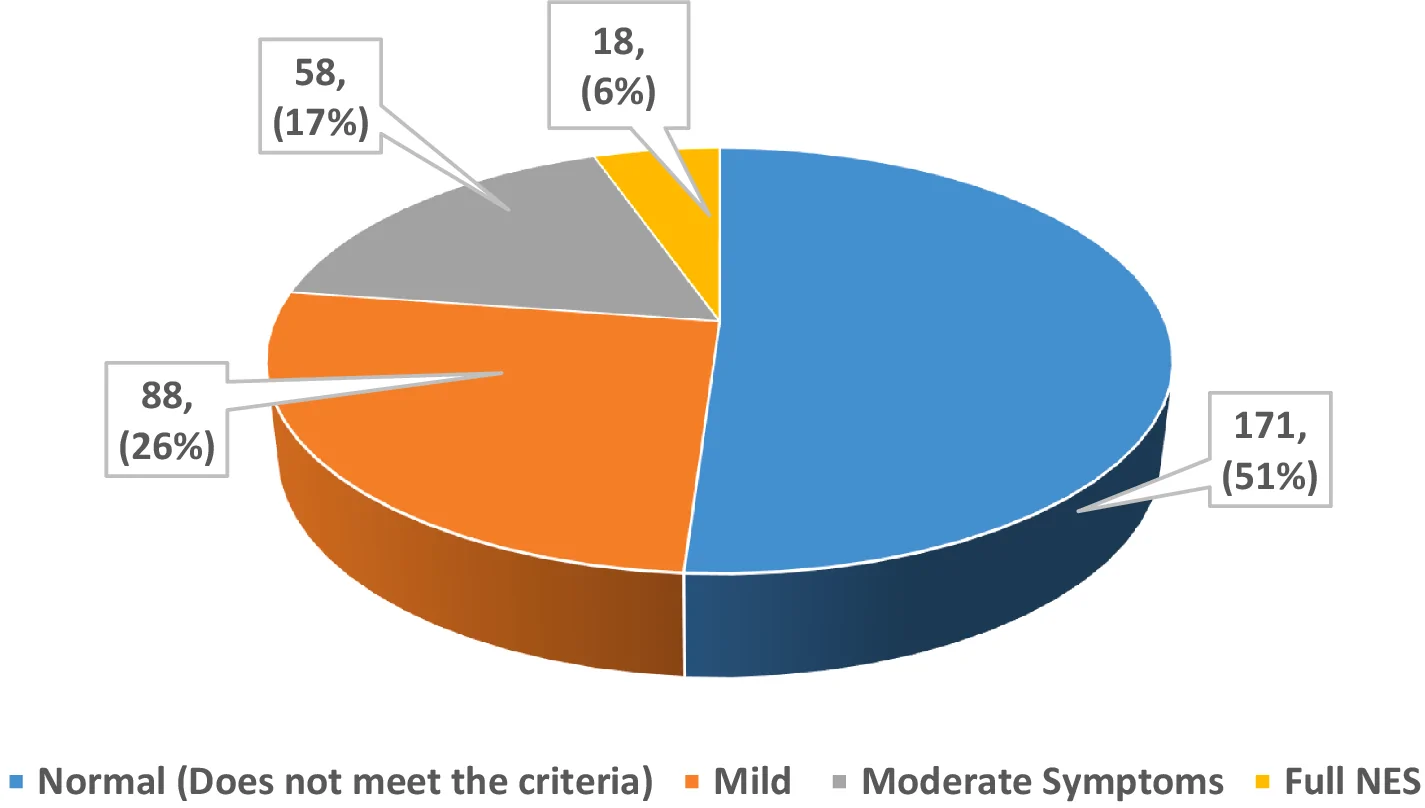
Distribution of night eating syndrome severity categories among participants.

**Table 2:** Distribution of sleep, circadian rhythms, and mood questionnaire subscale scores among participants.

SCRAM subscale	Mean ± SD
Good sleep (5–30)	18.2 ± 4.6
Morningness (5–30)	18.7 ± 4.0
Depressed mood (5–30)	16.6 ± 5.0

### 3.3 Circadian rhythm-related characteristics

Circadian-related, sleep-related, and mood-related characteristics were assessed using the SCRAM questionnaire. The average Good Sleep score was 18.2 ± 4.6, the average Morningness score was 18.7 ± 4.0, and the average Depressed Mood score was 16.6 ± 5.0. Comparisons of these SCRAM subscale scores across NES severity categories are presented below (see Table 3).

**Table 3:** Comparison of sleep, circadian rhythms, and mood questionnaire subscale scores across night eating syndrome severity categories.

Variable	Normal mean ± SD	Mild mean ± SD	Moderate mean ± SD	Full NES mean ± SD	Test statistic	*p*	η^2^ (95% CI)
Morningness	18.85 ± 3.21	20.45 ± 4.09	16.16 ± 4.60	17.61 ± 3.45	Welch *F* = 11.688	**<0.001**	0.127 (0.063–0.189)
Good sleep	18.37 ± 4.28	19.88 ± 3.93	15.74 ± 5.12	15.61 ± 5.15	*F* = 12.462	**<0.001**	0.101 (0.043–0.160)
Depressed mood	16.58 ± 4.90	15.88 ± 4.96	17.79 ± 4.92	16.78 ± 5.99	*F* = 1.736	0.159	0.015 (0.000–0.044)

Bold values indicate statistically significant results (*p* < 0.05).

### 3.4 Association between night eating syndrome and SCRAM subscale scores

SCRAM subscale scores were compared across NES severity categories using analysis of variance. Morningness scores differed significantly across severity categories (Welch’s *F* = 11.688, *p* < 0.001, η^2^ = 0.127, 95% CI: 0.063–0.189). Good Sleep scores also differed significantly across severity categories (*F* = 12.462, *p* < 0.001, η^2^ = 0.101, 95% CI: 0.043–0.160). In contrast, Depressed Mood scores did not differ significantly across severity categories (*F* = 1.736, *p* = 0.159, η^2^ = 0.015, 95% CI: 0.000–0.044) (see Table 4).

**Table 4:** *Post-hoc* pairwise comparisons of SCRAM subscale scores across NES severity categories.

SCRAM subscale	Pairwise comparison	Mean difference^1^	95% CI	Adjusted *p*-value
Morningness	Normal vs. Mild	−1.60	−2.90 to −0.30	**0.009**
	Normal vs. Moderate	2.70	0.99 to 4.41	**0.001**
	Normal vs. Full NES	1.24	−1.13 to 3.62	0.477
	Mild vs. Moderate	4.30	2.36 to 6.24	**<0.001**
	Mild vs. Full NES	2.84	0.32 to 5.36	**0.022**
	Moderate vs. Full NES	−1.46	−4.18 to 1.27	0.485
Good sleep	Normal vs. Mild	−1.51	−3.00 to −0.02	**0.046**
	Normal vs. Moderate	2.63	0.90 to 4.36	**<0.001**
	Normal vs. Full NES	2.76	−0.05 to 5.57	0.057
	Mild vs. Moderate	4.14	2.22 to 6.06	**<0.001**
	Mild vs. Full NES	4.27	1.33 to 7.21	**0.001**
	Moderate vs. Full NES	0.13	−2.93 to 3.19	>0.999
Depressed mood	Normal vs. Mild	0.70	−0.99 to 2.39	0.707
	Normal vs. Moderate	−1.21	−3.16 to 0.74	0.381
	Normal vs. Full NES	−0.20	−3.39 to 2.99	0.998
	Mild vs. Moderate	−1.91	−4.09 to 0.27	0.108
	Mild vs. Full NES	−0.90	−4.23 to 2.43	0.898
	Moderate vs. Full NES	1.01	−2.46 to 4.48	0.876

1 Mean differences were calculated as the first group minus the second group in each pairwise comparison. Bold values indicate statistically significant results (*p* < 0.05).

Games–Howell post-hoc comparisons showed that Morningness scores were significantly higher in the mild NES group than in the normal group (mean difference = −1.60, 95% CI: −2.90 to −0.30, adjusted *p* = 0.009) and significantly higher in the mild group than in the moderate group (mean difference = 4.30, 95% CI: 2.36 to 6.24, adjusted *p* < 0.001). The normal group also differed significantly from the moderate group (mean difference = 2.70, 95% CI: 0.99 to 4.41, adjusted *p* = 0.001), while the mild and full groups differed significantly (mean difference = 2.84, 95% CI: 0.32 to 5.36, adjusted *p* = 0.022).

For Good Sleep, the mild group had significantly higher scores than the normal group (mean difference = −1.51, 95% CI: −3.00 to −0.02, adjusted *p* = 0.046), while the normal group had significantly higher scores than the moderate group (mean difference = 2.63, 95% CI: 0.90 to 4.36, adjusted *p* < 0.001). The mild group also had significantly higher scores than both the moderate group (mean difference = 4.14, 95% CI: 2.22 to 6.06, adjusted *p* < 0.001) and the full group (mean difference = 4.27, 95% CI: 1.33 to 7.21, adjusted *p* = 0.001).

### 3.5 Multiple linear regression analysis of SCRAM subscale scores

Multiple linear regression analyses were conducted to examine the associations of NES status, sex, and BMI with Good Sleep, Morningness, and Depressed Mood scores (Table 5). NES status was significantly associated with Good Sleep scores (B = −2.711, *β* = −0.133, *p* = 0.017), but not with Morningness (B = −1.247, *β* = −0.071, *p* = 0.200) or Depressed Mood (B = 0.774, *β* = 0.035, *p* = 0.524). Sex was significantly associated with Morningness scores (B = 1.178, *β* = 0.117, *p* = 0.032), while BMI was significantly associated with Depressed Mood scores (B = −0.271, *β* = −0.165, *p* = 0.003). The regression models explained 1.7% of the variance in Good Sleep (*R*^2^ = 0.017, adjusted *R*^2^ = 0.009, *p* = 0.120), 1.9% in Morningness (*R*^2^ = 0.019, adjusted *R*^2^ = 0.010, *p* = 0.099), and 3.1% in Depressed Mood (*R*^2^ = 0.031, adjusted *R*^2^ = 0.022, *p* = 0.016).

**Table 5:** Multiple linear regression models for SCRAM subscales adjusted for NES status, sex, and BMI.

Predictor	Good Sleep B	*β*	*p*-value	Morningness B	*β*	*p*-value	Depressed Mood B	*β*	*p*-value
NES status	−2.711	−0.133	0.017	−1.247	−0.071	0.200	0.774	0.035	0.524
Sex	0.064	0.005	0.920	1.178	0.117	0.032	0.677	0.054	0.324
BMI	0.009	0.006	0.919	0.041	0.031	0.570	−0.271	−0.165	0.003

Model statistics				
Outcome	*R* ^2^	Adjusted *R*^2^	F-statistic	Model p-value
Good sleep	0.017	0.009	1.908	0.120
Morningness	0.019	0.010	2.137	0.099
Depressed mood	0.031	0.022	3.530	**0.016**

NES status was coded as 0 = No NES and 1 = NES, with No NES as the reference category. Sex was coded as 0 = Male and 1 = Female, with Male as the reference category. Bold values indicate statistically significant results (*p* < 0.05).

### 3.6 Association between BMI categories and night eating syndrome

The association between BMI categories and NES status was assessed using the Fisher–Freeman–Halton exact test because of sparse expected cell counts (Table 6). A statistically significant association was observed between BMI category and NES status (exact test, *p* = 0.002). The proportion of participants meeting the NEDQ criteria for NES was 4.2% among underweight participants, 2.7% among those with normal weight, 25.0% among overweight participants, and 23.1% among participants with obesity class I. None of the participants in the obesity class II category met the NEDQ criteria for NES.

**Table 6:** Association between BMI categories and night eating syndrome status.

BMI Category	No NES *n* (%)	NES *n* (%)	Exact test *p*-value
Underweight	184 (95.8)	8 (4.2)	
Normal weight	107 (97.3)	3 (2.7)	
Overweight	12 (75.0)	4 (25.0)	
Obese class I	10 (76.3)	3 (23.1)	
Obese class II	4 (100.0)	0 (0.0)	
Total	317 (94.6)	18 (6)	0.002

Fisher–Freeman–Halton exact test was used because of sparse expected cell counts.

BMI category was also significantly associated with NES severity (Table 7). Pearson’s chi-square test showed a significant association between BMI category and NES severity (χ^2^ = 34.95, df = 12, *p* < 0.001). The likelihood-ratio chi-square test also indicated a significant association (χ^2^ = 28.16, df = 12, *p* = 0.005).

**Table 7:** Association between BMI category and NES severity.

BMI category	Normal *n* (%)	Mild *n* (%)	Moderate *n* (%)	Full NES *n* (%)	Total
Underweight	101 (52.6)	55 (28.6)	28 (14.6)	8 (4.2)	192
Normal weight	54 (49.1)	25 (22.7)	28 (25.5)	3 (2.7)	110
Overweight	8 (50.0)	3 (18.8)	1 (6.3)	4 (25.0)	16
Obese class I	4 (30.8)	5 (38.5)	1 (7.7)	3 (23.1)	13
Obese class II	4 (100.0)	0 (0.0)	0 (0.0)	0 (0.0)	4
Total	171	88	58	18	335

Pearson *χ*^2^ = 34.95, df = 12, *p* < 0.001; likelihood-ratio *χ*^2^ = 28.16, df = 12, *p* = 0.005.

## 4 Discussion

This study investigated NES and circadian-related characteristics among 335 undergraduate students in Rawalpindi and Islamabad. The average age of participants was 21.97 ± 1.15 years, thus representing a sample group of young adults in universities. Most of the participants were female, unmarried, living with family, and pursuing undergraduate courses related to health sciences. The prevalence of NES in the current study was found to be 6% of the total number of participants. Severity-based analysis showed that 26.3% had symptoms of mild NES and 17.3% showed symptoms of moderate NES, indicating that night-eating-related symptoms were present in a considerable proportion of students even without meeting full NEDQ criteria for NES.

The prevalence of NES obtained in the current study is consistent with findings from previous studies carried out among university students. For instance, Runfola et al. found the prevalence of NES in university students in the United States to be between 2.9 and 4.2% excluding those with binge eating disorders (33). In the same way, Hao et al. (41) estimated the prevalence of 2.8% in the case of Chinese university students. This may be related to the use of similar assessment tools and the comparable age of the populations studied. At the same time, prevalence estimates varied across Middle Eastern and South Asian studies. Thus, for example, Hamdan et al. (29) found the prevalence rate of 29.7%. Sahrin et al. (31) reported 16.6% among Bangladeshi university students, and Elsahoryi et al. (34) reported a higher frequency of night eating symptoms among Jordanian university students. The variations can be accounted for by different diagnostic tools, cut-off points, participant characteristics, dietary habits in cultures, psychosocial stresses, hostel living conditions, and lifestyle habits.

In Pakistan, previous studies have also reported variation in the occurrence of Night Eating Syndrome and related night eating symptoms. Batra et al. (38) reported a prevalence of 14.4% in Karachi, while another study among medical students in Karachi reported a higher frequency of night eating symptoms and a significant association with depression (42). These differences may reflect variation in assessment methods, participant characteristics, and study settings.

This is supported by research done before on university students who are known to exhibit irregularity in sleep–wake timing and patterns, stress, and lifestyle variation due to variations in sleep–wake timing and lifestyle patterns. Phillips et al. (24) noted that irregularity in sleep–wake timing and delayed timing is common amongst university students and could disrupt their circadian rhythms. Likewise, Hershner and Chervin (23) noted that academic pressures, social life, and lifestyle choices cause inadequate and irregular sleep in college students. Additionally, Roenneberg et al. (25) defined social jet lag as a difference in biological and social timing, and is applicable to young adults. Therefore, the results of the current SCRAM test can be contextualized based on student lifestyles and sleep–wake timing variations related to academic scheduling and circadian-related patterns.

Severity-based analysis provided further information on the relationship between night eating symptoms and circadian-related and sleep-related characteristics. There was a significant difference in the scores of Morningness and Good Sleep in different groups of NES severity; however, there were no significant differences in the score of Depressed Mood between different groups of NES severity. These findings suggest that characteristics related to diurnal preference and sleep may be more evident when night eating symptoms are considered by severity.

The significant differences in Morningness scores across the severity categories are noteworthy. The low values of Morningness scores in the moderate and full NES severity categories may indicate a tendency toward a later diurnal preference among students with higher night eating symptom severity. It confirms the findings from previous studies on eveningness, delayed behavioral phase, night eating symptoms, and irregularity of meal timings. Karakaya and Arıbaş (43) found that chronotype preference and delayed circadian rhythms were related to night eating symptoms among university students. Even though the current study does not include classification of chronotype, the Morningness subscale provides useful information regarding diurnal preference. In the present study, Morningness should therefore be interpreted as an indicator of individual differences in diurnal preference rather than a direct measure of circadian rhythm disruption.

Moreover, the scores for Good Sleep also were found to be significantly different in accordance with night eating severity. As it can be seen from the data presented, lower scores on the Good Sleep subscale in the moderate and full NES groups indicate poorer sleep-related characteristics among participants with more pronounced night eating symptoms. Such results coincide with those of Alwafa et al. (27) who found the connection between Night Eating Syndrome and poor sleep quality among university students. Furthermore, Elsahoryi et al. (34) confirmed the link between the Night Eating Syndrome and sleep disturbances. Thus, the current research provides further support to the hypothesis that sleep-related characteristics were associated with the degree of severity of NES.

Depressed Mood scores did not differ significantly across the NES severity groups. This is somewhat different from what was reported in earlier studies on the relationship between NES and depressive symptoms. There could be several reasons for this finding, including culture-specificity in the reporting of mood, non-clinical sample of the research, small number of participants with full NES, or the use of SCRAM as a measure of mood-related characteristics rather than a diagnostic measure of depression. Hence, the results should be considered cautiously.

Further information about these relationships was obtained from multiple linear regression analyses adjusting for NES status, sex, and BMI. NES status was significantly associated with Good Sleep scores but was not significantly associated with Morningness or Depressed Mood scores. Sex was significantly associated with Morningness, while BMI was significantly associated with Depressed Mood. However, the models explained only a small proportion of the variance in SCRAM subscale scores, indicating that these predictors accounted for limited variability in the outcomes.

A significant association was observed between BMI category and both NES status and NES severity. The proportion of participants meeting the NEDQ criteria for NES varied across BMI categories, with higher proportions observed among overweight and obesity class I participants. However, these findings should be interpreted cautiously because some BMI categories contained very small numbers of participants, particularly the obesity class II category, in which no participant met the NEDQ criteria for NES. The cross-sectional design also does not allow determination of the direction of the association or whether shared behavioral factors contributed to the observed findings.

The findings have potential public health implications for university settings. Given the presence of mild and moderate night eating symptoms in a considerable proportion of students, university-based health promotion strategies may consider addressing sleep hygiene, regular meal timing, regular sleep–wake patterns, and awareness of night eating symptoms. Early identification of night eating behaviors may also help inform appropriate support and referral where needed. However, these recommendations were not directly evaluated in the present study and should therefore be considered as potential areas for future research and health promotion planning.

There are some weaknesses that can be noted in this study. Because of the cross-sectional design, causal relationships between NES and circadian-related characteristics cannot be established. The data was gathered through self-administered questionnaires; therefore, there might have been the influence of recall bias and social desirability bias. Self-reported height and weight data can have affected the classification of body mass index. In addition, the number of participants with full NES was rather small, and some BMI categories contained few participants. These sparse cells may have reduced the statistical power and should be considered when interpreting the BMI-related associations. However, it is worth noting that the factors such as academic stress, screen time, sleep duration, chronotype, dietary intake, and meal times were not considered. Furthermore, participants were recruited from selected universities in Rawalpindi and Islamabad and were limited to Allied Health Sciences students in the fourth to eighth semesters. Therefore, the findings may not be generalizable to students from other academic disciplines, semesters, universities, or student populations.

Overall, the current study demonstrates the presence of Night Eating Syndrome and related symptoms among undergraduate students in selected universities of Rawalpindi and Islamabad. Severity-based analysis indicated significant differences in Morningness and Good Sleep scores across NES severity categories, whereas Depressed Mood did not differ significantly. The findings suggest that sleep-related characteristics and diurnal preference may be more closely associated with the severity of night eating symptoms than mood-related characteristics. However, these findings should be interpreted as associations with circadian-related behavioral characteristics rather than evidence of circadian rhythm disruption. The high proportion of female participants may limit the generalizability of the findings, particularly for sex-related associations.

## 5 Conclusion

Among university students in Rawalpindi and Islamabad, mild and moderate night eating symptoms were more common than participants meeting the full NEDQ criteria for Night Eating Syndrome. Increasing severity of night eating symptoms was associated with differences in Morningness and Good Sleep scores, while Depressed Mood scores did not differ significantly across severity categories. These findings suggest that night eating symptoms are associated with sleep-related characteristics and diurnal preference and may inform future university-based research and health promotion planning related to sleep hygiene, regular meal timing, and early identification of night eating behaviors.

## Data Availability

The raw data supporting the conclusions of this article will be made available by the authors, without undue reservation.
